# Immune escape of *Staphylococcus aureus* mediated by osteocyte lacuna-canalicular network leads to persistent and uncured bone infection

**DOI:** 10.3389/fcimb.2025.1592086

**Published:** 2025-06-02

**Authors:** Zhigang Rong, Xiaozhen Chen, Leilei Qin, Xiaohua Wang, Fei Luo, Quanming Zou, Hao Zeng

**Affiliations:** ^1^ Department of Orthopaedics, Southwest Hospital, Third Military Medical University (Army Medical University), Chongqing, China; ^2^ National Engineering Research Center of Immunological Products, Third Military Medical University (Army Medical University), Chongqing, China; ^3^ Institute of Cancer, Xinqiao Hospital, Third Military Medical University (Army Medical University), Chongqing, China; ^4^ Department of Orthopaedics, The First Affiliated Hospital of Chongqing Medical University, Chongqing, China

**Keywords:** *Staphylococcus aureus*, osteocyte lacuno-canalicular network, immune escape, bone infection, chronic osteomyelitis

## Abstract

Bone infections, specifically chronic osteomyelitis, are characterized by recurrent episodes. They are considered intractable clinical diseases as they require protracted and difficult-to-cure courses. *Staphylococcus aureus* (*S. aureus*) is the most common pathogen responsible for bone infections and has high destruction rates. Previous literature has indicated that during *S. aureus* osteomyelitis, immune evasion mainly involves three mechanisms: biofilm formation, intracellular infection, and abscess formation. However, recently, it was observed that *S. aureus* can enter and persist for a long time in the Osteocyte lacuno-canalicular network (OLCN), a bone microstructure. Furthermore, it has been found to successfully evade the host’s immune system *via* natural physical barriers, chemical properties, and bone microstructure’s immune escape mechanisms. Therefore, *S. aureus* bone infections are more difficult to cure than soft-tissue infections. Currently, there are only a few studies on OLCN invasion by *S. aureus*, and the clinical evidence is not sufficient. Therefore, this review aimed to combine relevant published literature on the OLCN-mediated immune escape of *S. aureus* to elaborate on the pathological mechanisms associated with protracted and difficult-to-cure bone infections. The findings will provide a scientific basis and theoretical foundation for future comprehensive analysis of how *S. aureus* invades OLCN and novel treatment strategies for bone infections.

## 1 Introduction

Chronic osteomyelitis (COM) is a chronic infection of bone primarily caused by pathogenic microorganisms disseminating *via* hematogenous or exogenous routes. COM is manifested in bones and surrounding soft tissues, usually after months to years of continuous infection. Furthermore, it is mostly diagnosed secondary to neglected or incompletely treated hematogenous osteomyelitis and is a common complication observed after open fractures and orthopedic-related surgeries. COM is associated with local bone tissue necrosis and abscess formation; therefore, it is characterized by long-course complex infections with a high recurrence rate. In severe cases, it can cause bone defects and even become a lifelong disease. Reports have indicated that COM can relapse 80 years after the primary onset (Kremers et al., 2015). The clinical treatment of COM is difficult and costly, which significantly impacts patients’ quality of life and the healthcare system and has become a major challenge for orthopedic surgeons (Wu et al., 2019; Yousaf et al., 2021).

Chronic osteomyelitis predominantly occurs after fractures and internal fixation implantation surgeries. However, in rare cases, it is also caused by lower-limb ischemic ulcers due to diabetes, sickle-cell disease, and malnutrition (Jiang et al., 2024). The subtype classification of COM includes implant-related osteomyelitis [including periprosthetic joint infection (PJI) and instrumented spinal infection], fracture-related infection, acute hematogenous osteomyelitis, diabetic foot infection, septic arthritis, congenital spinal osteomyelitis, *etc.* (Masters et al., 2022). Its primary characteristics include the persistent presence of microorganisms, low-grade inflammation, sequestra (bone fragments), and sinus tract formation, which differentiates it from acute osteomyelitis (Caputo et al., 1994). Recurrence at the same site accompanied by fever is an obvious sign of COM. Moreover, persistent clinical symptoms for > 10 days have been linked with the formation of sequestra and the development of COM (Norden et al., 1992).

Various microorganisms have been associated with bone infections. Some common microorganisms causing chronic bone infections include *Staphylococcus aureus (S. aureus*), *coagulase-negative staphylococci* (CoNS) (such as *Staphylococcus epidermidis*), *Streptococcus* spp., *Enterococcus* spp., *Diphtheroids*, Gram-negative bacteria, *Pseudomonas* spp., and *Enterobacteriaceae* spp. Of these, *S. aureus* is the most common, prevalent, and destructive pathogen related to bone infections (Masters et al., 2022). This Gram-positive bacterium can infect about every type of human tissue and cause asymptomatic skin colonization to life-threatening diseases. Furthermore, *S. aureus* is specifically pathogenic in bone infections because it can invade, colonize, and grow within bones (Masters et al., 2022). Moreover, its virulence factors can disrupt the host’s immune defenses, thereby causing bone destruction. For instance, *S. aureus* protein A (SpA), an extracellular and cell-binding protein, can induce severe inflammatory responses (Kumar et al., 2007), inhibit osteogenesis and promote osteoclastogenesis (Jin et al., 2013). Similarly, > 50% of bone infections are caused by methicillin-resistant *S. aureus* (MRSA), which is difficult to treat (Gallarate et al., 2021).

Chronic bone infections are primarily controlled using the following 6 measures: thorough debridement, local stabilization, dead space obliteration, adequate drainage, effective coverage, as well as applying local and systemic sensitive antibiotics. However, these clinical treatment strategies face 2 challenges. First, antibiotics have a low bone tissue diffusion rate, and although administered intravenously, the required plasma concentration and tissue penetration are difficult to achieve (Fantoni et al., 2019). Furthermore, microorganisms have evolved various mechanisms to effectively evade the host’s innate and adaptive immune attacks and continuously develop antibiotic resistance for persistent colonization in the host (Qin et al., 2024), which increases the challenges of COM treatment. Secondly, thorough surgical debridement of inflammatory tissues, sinus tracts, scar tissues, infected granulation tissues, medullary cavity abscesses, sclerotic bone, sequestra, *etc.*, is crucial for COM treatment. Most current studies suggest that it is necessary to expand the debridement region, and tissue that is infected or might be infected, as well as the surrounding soft tissues, should be removed as much as possible (Simpson et al., 2001; Hevroni and Koplewitz, 2007; Hogan et al., 2013). However, the accurate identification of the infection boundary is difficult to delineate during surgery for thorough debridement. Therefore, even after antibiotic treatment and surgical debridement, the treatment failure rate is 20% (Conterno and Turchi, 2013), and the postsurgical reinfection rate is 33% (Azzam et al., 2009; Rosas et al., 2017).

**Figure 1:**
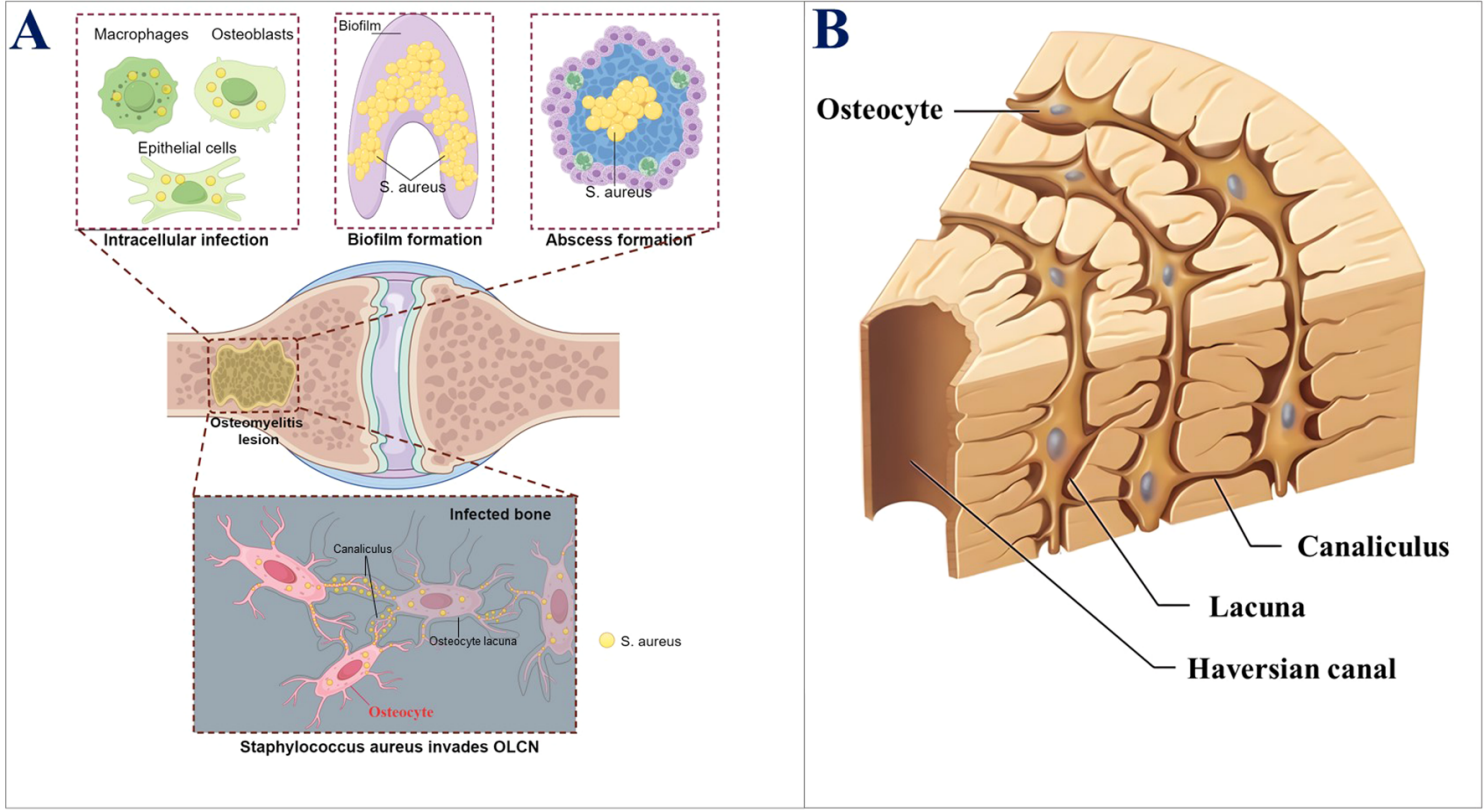
**(A)** Key mechanisms of the persistence of S. aureus in bone infections. **(B)** Schematic diagram of the three-dimensional structure of OLCN.

Several studies have indicated that the following mechanisms are responsible for immune evasion and *S. aureus* persistence during COM: Biofilm formation (Ricciardi et al., 2018; Masters et al., 2019), Intracellular infection (Ellington et al., 2003; Bosse et al., 2005; Sendi et al., 2006; Josse et al., 2015; Yang et al., 2018; Krauss et al., 2019; Alder et al., 2020; Roper et al., 2020), Staphylococcal abscess communities (SACs) (Carek et al., 2001; Cheng et al., 2011; Kobayashi et al., 2015; Malachowa et al., 2016; Kavanagh et al., 2018). These mechanisms have been comprehensively studied and, therefore, will not be discussed in this paper. The *S. aureus* invasion of the Osteocyte lacuno-canalicular network (OLCN) *via* a novel pathway for bacterial persistence and immune evasion can explain the long-term bacterial persistence and treatment failure in COM (de Mesy Bentley et al., 2017; de Mesy Bentley et al., 2018) (**Figure 1A**). However, this pathway has not been systematically investigated, and comprehensive research is needed to determine better COM treatment. Therefore, this paper reviewed this pathway to provide the theoretical foundation for exploring the mechanism of *S. aureus* invading the OLCN and developing targeted treatment strategies in the future.

## 2 Osteocyte lacuno-canalicular network

Osteocytes are the primary cells in the bone matrix (Parfitt, 1977). Furthermore, they are the most mature and terminally differentiated cells within the osteoblast lineage. Moreover, they are found embedded in the lacunae (or bone lacunae) of the hard extracellular matrix (ECM). Lacunae are the spaces that contain a single osteocyte. The osteocyte has a flat and oval-shaped cell body, a slightly darker colored nucleus, and basophilic cytoplasm, with observable mitochondria and Golgi apparatus. Moreover, glycogen granules and lipid droplets can be revealed by special staining. Osteocytes have numerous slender processes, which extend into the small canals (called bone canaliculi) around the bone lacunae. That is, the bone canaliculi connect adjacent bone lacunae, which are channels containing the cytoplasmic processes of osteocytes (Yu et al., 2020). The most prominent feature of osteocytes is their cell processes, which extend within the bone canaliculi and connect with adjacent osteocytes. These structures allow osteocytes in the deep part to connect and communicate with the cell processes and bone canaliculi near the bone surface. These cell processes are connected *via* gap junctions (Doty, 1981; Shapiro, 1997). This huge three-dimensional network structure is called an OLCN (Burger and Klein-Nulend, 1999) (**Figure 1B**). The osteocyte OLCN has various crucial functions, such as mechanical sensing, bone remodeling balance, and homeostasis of body mineral metabolism (Repp et al., 2017). The bone canaliculi are transport channels for blood and nutrients within the bone. They supply nutrients and oxygen to the bone and are the only route for the bone tissue to communicate with the outside. Osteocytes in the bone matrix maintain the ecological balance within the bone by transporting nutrients and metabolic products *via* bone canaliculi and also provide a signal transmission pathway within the bone. Therefore, it was inferred that OLCN provides a suitable living environment, such as sufficient nutrients, oxygen, and an appropriate pH, for the invading *S. aureus*, enabling it to survive persistently in the OLCN and evade the immune system, which might explain the protracted course of chronic bone infections. The relationship between the OLCN environment and *S. aureus* survival warrants further research.

## 3 Clinical evidence of *S. aureus* invading the OLCN

It has been determined that most COM cases involve *S. aureus* infections (either single-microbe or multi-microbe). Despite aggressive surgical debridement and antibiotic treatment, recurrence remains common. The 2023 International Consensus Conference on Musculoskeletal Infections emphasized that eradicating residual bacteria is crucial for treating implant-related osteomyelitis (Jennings et al., 2024). As mentioned above, the invasion of *S. aureus* into the OLCN may render it a challenging pathogen in bone infections. This newly discovered unique immune evasion phenomenon of *S. aureus* can be initially traced back to a clinical case reported in 2018. This report described a patient with an infected diabetic foot ulcer complicated by COM due to *S. aureus*. Gram-staining confirmed the presence of Gram-positive bacteria in a fan-shaped pattern in the bone tissue adjacent to the bone marrow. Transmission electron microscopy (TEM) was used to describe that in the sub-micron OLCN of the amputated bone tissue, *S. aureus* transformed from spherical cocci into rod-shaped bacteria. This was the first evidence of the transformation of *S. aureus* and its invasion into the OLCN in human bones, supporting a new mechanism for the persistent existence of *S. aureus* in the pathogenesis of COM (de Mesy Bentley et al., 2018).

Subsequently, Louise Kruse Jensen et al. also studied two patients with COM (one with diabetic foot osteomyelitis and one with fracture-related infection). The microbiological test results of all patients were positive for Staphylococcus. Meanwhile, the clinical relevance of bacterial invasion into the sub-micron OLCN in bone tissue was confirmed through testing. Based on immunohistochemistry and electron microscopy, *S. aureus* was identified in the OLCN of all patients. These findings solidified that bacterial OLCN invasion is a clinically relevant part of the disease biology of osteomyelitis (Jensen et al., 2023), especially regarding osteomyelitis recurrence. The literature mentioned above represents the only conclusive clinical evidence of *S. aureus* invading OLCN. It provides new insights into immune evasion and persistence during *S. aureus* osteomyelitis and indicates a new direction for future research, thus being of great value. However, they merely point out the real-world phenomenon of *S. aureus* invading OLCN during osteomyelitis without further exploring the reasons for its invasion, the specific biological processes, and mechanisms in combination with clinical cases. Many subsequent studies mainly rely on *in-vitro* platforms and animal models, which may differ from clinical cases and cannot be fully used to guide future clinical practice. Therefore, continued in-depth research in combination with cases of chronic bone infection caused by *S. aureus* may also be one of the most promising research directions in the future.

## 4 The OLCN invading mechanism of *S. aureus*

For chronic bone infection, the extensive and thorough debridement, irrigation, removal of all implants in traditional revision surgeries, and systemic antibiotic therapy are effective against planktonic bacteria, SACs, and surface biofilms; however, these measures remain ineffective against the bacteria in OLCN (Ren et al., 2022; Ren et al., 2023). *S. aureus* can invade OLCN by various mechanisms, making chronic bone infections more difficult to treat. Firstly, the OLCN is located deep within the bone cortex in the bone mineral matrix, an environment that restricts immune-cell-mediated immune surveillance, making it completely immune to attacks from immune cells. Further, osteocytes have a long life and can survive in the body for decades (Prideaux et al., 2016). These factors make OLCN a perfect site for the persistent survival of *S. aureus*. Secondly, bacteria may survive for years by dissolving the surrounding bone mineral matrix as a source of nutrients. Lastly, the depth to which *S. aureus* invades the OLCN remains unclear; however, it might be a primary factor responsible for the failure of surgical removal of bone infection foci. In summary, theoretically, amputation may be the only treatment approach to eradicate *S. aureus* in the OLCN of living bone tissue (Masters et al., 2020). Therefore, a comprehensive investigation of the mechanisms by which *S. aureus* invades the OLCN is required for the development of novel treatment strategies and for reducing the recurrence and disability rate associated with chronic bone infections.

Bone canaliculi have about 0.5 μm diameter and connect osteocyte lacunae (You et al., 2004); however, the diameter of most clinically relevant bacteria is much larger (for example, *S. aureus* is 1 μm in diameter). Therefore, it is generally believed that bacteria cannot enter the OLCN, and indeed, the OLCN invasion by *S. aureus* is challenged by morphological variation. However, the literature suggests that for OLCN invasion, *S. aureus* transforms from cocci to rod-shaped bacteria with a diameter less than half its original size (de Mesy Bentley et al., 2017; Yu et al., 2020). Furthermore, since *S. aureus* has no flagella, its invasion of OLCN is also contrary to the consensus that *S. aureus* is a non-motile coccus (Masters et al., 2022). These findings have attracted many scholars to study the mechanism by which *S. aureus* enters the OLCN after morphological variation.

De Mesy Bentley et al. performed transmission electron microscopy (TEM) and revealed that *S. aureus* (UAMS-1, USA300 LAC) exists as a single cocci and sub-micron rod-shaped bacteria in the canals of the living cortical bone of mice and forms biofilms in the osteocyte compartments. They also found that the deformed bacteria can enter the canals *via* asymmetric binary fission, proliferate along the osteocyte lacunae edges, and migrate into the lacunae’s interior (de Mesy Bentley et al., 2017). Another study suggests that *S. aureus* invades OLCN by stimulating its cell-wall synthesis mechanism and surface adhesins under the guidance of durotaxis (Raimon et al., 2016) and haptotaxis (Hsu et al., 2005), respectively. To identify the virulence genes responsible for haptotaxis and durotaxis, an *in-vitro* platform model, the microfluidic silicon membrane-canal array (μSiM-CA), was developed (de Mesy Bentley et al., 2017; Elysia et al., 2019). This model mimics canaliculi’s physiological dimensions and determines the genes required for *S. aureus* to invade the canaliculi. It has been observed that the Agr quorum-sensing system may not be necessary for the *in-vitro* transmission of *S. aureus* (UAMS-1) through 0.5-micrometer nanopores (Elysia et al., 2019). Furthermore, in a murine implant-related osteomyelitis model, Agr gene knockdown *S. aureus* [UAMS-1 agr-null strain (UAMS-1*Δagr.:tetM*)] invaded the sub-micron canal network of bone (Gowrishankar et al., 2019). Penicillin-binding proteins 3 and 4 (PBP3 and PBP4) genes encode the non-essential cell-wall transpeptidases, PBP3 and PBP4, respectively, which function in the final stage of cell-wall synthesis (Costa et al., 2018). Elysia A Masters et al. employed the μSiM-CA platform model to screen the *S. aureus* transposon-insertion mutant library (Elysia et al., 2019; Masters et al., 2020) and indicated that PBP4 gene deletion significantly inhibited the *in-vitro* transmission of *S. aureus* (USA300) through nanopores, inhibited *S. aureus* invasion of OLCN, and reduced the degree of bone loss at the infection site in a murine model of implant-related osteomyelitis. Moreover, they investigated the *in-vitro* nanopore transmission and *in-vivo* osteomyelitis pathogenesis of selected *S. aureus* cell wall synthesis mutants. The results of the *in-vitro* study showed that the deletion of cell-wall synthesis mutants [PBP3, autolysin (Atl)] and surface adhesin mutants [clumping factor A (ClfA) and SasC] inhibited *S. aureus* transmission through nanopores. Furthermore, in a murine implant-related osteomyelitis model, PBP3 and Atl deletion [USA300 pbp3-null (*Δ*pbp3) and atl null (*Δ*atl)] reduced the loosening of infected implants and the formation of *S. aureus* abscesses in the bone marrow cavity, while the deletion of surface adhesins had no significant difference. TEM imaging revealed that PBP3 was the only mutant gene associated with reduced *in vivo* sub-micron canal invasion (Elysia et al., 2021). These *in-vitro* and *in-vivo* analyses confirmed that PBP3 and PBP4 are key genes for *S. aureus’* sub-micron transmission and OLCN invasion (Masters et al., 2020). Therefore, inhibiting PBP3 and/or PBP4 may prevent *S. aureus* invasion of OLCN, and their inhibitors can be used as adjuvants in the antibacterial treatment of *S. aureus* osteomyelitis (Masters et al., 2022). Overall, the cell wall synthases of *S. aureus* are crucial for *in vivo* OLCN invasion and osteomyelitis pathogenesis.

The survival of bacteria requires cell wall protection, and the main structural unit of the bacterial cell wall is peptidoglycan (also called mucopetide), a unique component that is composed of sugars and amino acids. Peptidoglycan is a derivative of heteropolysaccharide and is considered “bricks,” which are crucial for maintaining cell morphology, integrity, mechanical strength, and the survival of bacteria (Vollmer et al., 2008a; Silhavy et al., 2010; Turner et al., 2014). However, peptidoglycan alone is insufficient, and to build a wall using these bricks, penicillin-binding proteins (PBPs) are required, which act as “cement.” The PBPs can cross-link peptidoglycan into a complete cell wall and are involved in the final assembly stage of peptidoglycan into the bacterial cell wall. The repair of *S. aureus* cell wall has four endogenous PBPs (PBP1, PBP2, PBP3, PBP4). Of these, only PBP1 and PBP2 are required for peptidoglycan synthesis, as they can perform all the transpeptidase (side-chain-linking) functions required for cell growth and division (Pinho et al., 2001; Pereira et al., 2007; Eric et al., 2008; Katarzyna et al., 2022). PBP3 and PBP4 are non-essential, monofunctional PBPs with only transpeptidase activity (Pinho et al., 2000; Scheffers and Pinho, 2005). PBP3 is a class B high-molecular-weight PBP, and its role in the S. aureus cell cycle remains elusive (Yoshida et al., 2012; Kylväjä et al., 2016). PBP4 also has a low molecular weight and is the only PBP responsible for the extensive peptidoglycan cross-linking in the *S. aureus* cell wall (Navratna et al., 2010). Peptidoglycan hydrolases balance the function of PBPs by degrading the cell wall during growth and division. Atl is the major peptidoglycan hydrolase of *S. aureus*, which consists of two subunits: amidase (Amd) and glucosaminidase (Gmd) (Vollmer et al., 2008b). It participates in cell wall hydrolysis during cell division (Clarke and Foster, 2006; Vollmer et al., 2008b) and also binds ECM ligands as a surface adhesin. Therefore, it was proposed that Atl may be involved in haptotaxis and durotaxis functions (Clarke and Foster, 2006; Bose et al., 2012; Hirschhausen et al., 2012; Schlesier et al., 2020), which may be related to the invasion of OLCN by *S. aureus* (Elysia et al., 2021).

Theoretically, *S. aureus* must deform and divide into smaller-size/diameter cells to invade OLCN. Bacterial cell division (mainly binary fission) is a relatively complex process, where during DNA molecule separation, the cell membrane and wall grow inward to form a septum, dividing the cytoplasm into two halves. Other than peptidoglycan (the main structural component of the septum) and PBPs, the cell wall of *S. aureus* contains other crucial components, including tyrosine, β-glucosidase, lactic acid, and lactate dehydrogenase. These components coordinate with each other to form special cell wall structures and perform various functions, such as cell protection, cell shape maintenance, and drug resistance. Furthermore, they also modulate the formation of the septum and cell division in terms of space and time. An in-depth evaluation of the *S. aureus* cell wall components will help understand their biological characteristics and application value.

Currently, the research on the mechanism of *S. aureus* invading OLCN is mostly based on *in-vitro* simulation platforms and murine osteomyelitis model experiments, and the OLCN invading mechanism of *S. aureus* in clinical chronic bone infection patients remains undetermined. Therefore, it is crucial to research clinical cases to comprehensively understand the underlying mechanism and better prevent the occurrence and development of chronic bone infections.

## 5 Treatments for *S. aureus* invasion of OLCN

**Figure 2:**
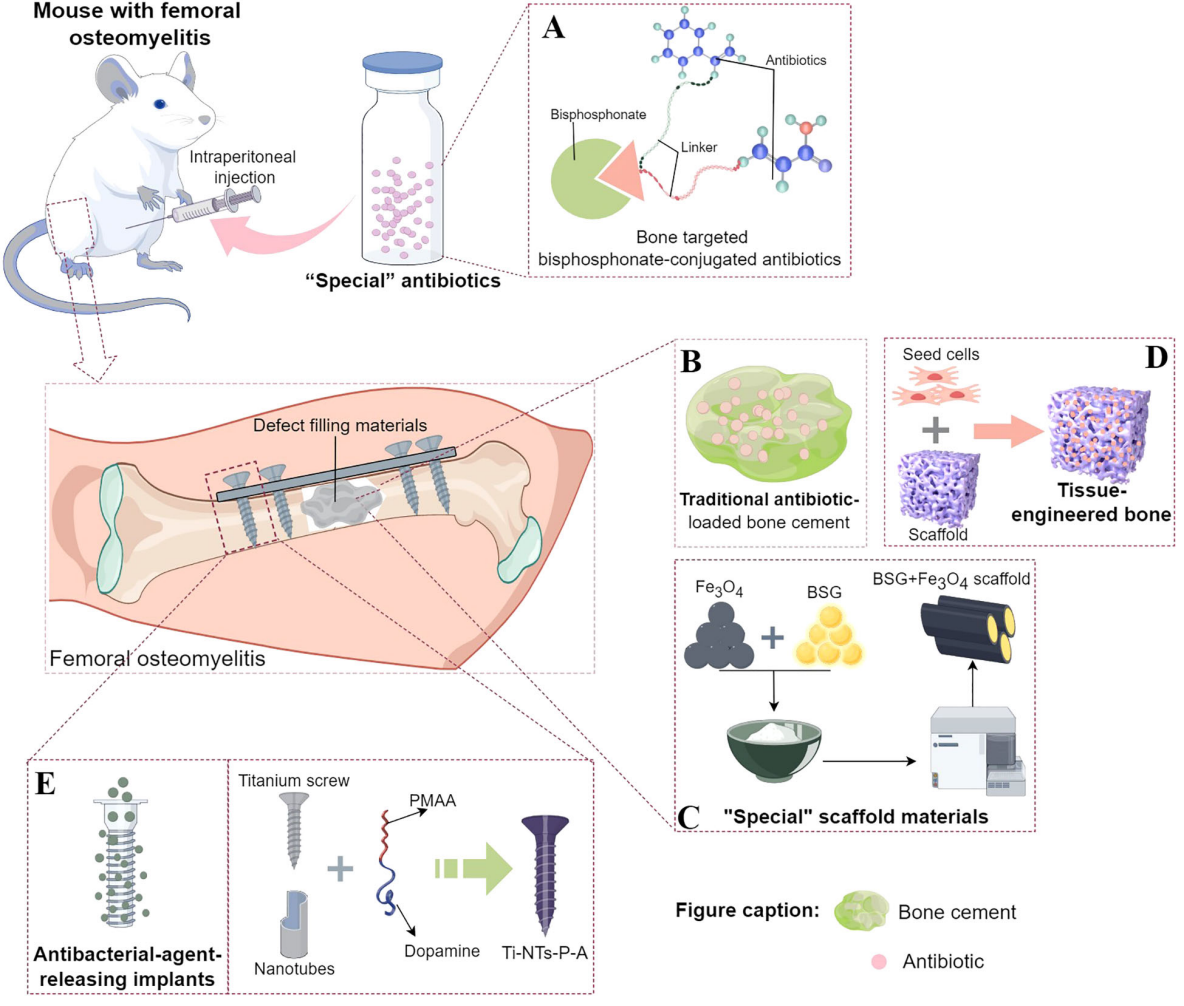
Existing treatment strategies targeting the immune escape mechanism of Staphylococcus aureus invasion into the OLCN. **(A)** The composition of “special” antibiotic, bisphosphonate-conjugated antibiotics. Part of the design was cited from Reference 74 and redrawn. **(B)** The basic structure of traditional antibiotic-loaded bone cement. **(C)** A schematic diagram taking the “special” scaffold material composed of borosilicate bioactive glass (BSG) + Fe_3_O_4_ magnetic nanoparticles as an example. Part of the design was cited from Reference 77 and redrawn. **(D)** The composition of tissue - engineered bone, including seed cells and scaffolds. **(E)** A schematic diagram of the implant material capable of sustained - release of antibacterial agents, taking Ti - NTs - P - A as an example. Part of the design was cited from Reference 90 and redrawn.

Due to the lack of research on the colonization of *S. aureus* in the OLCN and the evidence of clinical efficacy, the use of local antibiotics to treat bone infections is being questioned. Researchers have searched PubMed using “osteocyte lacuno-canalicular network” and/or “OLCN” as keywords, to investigate the treatment of *S. aureus* invasion of the OLCN, which revealed the following three aspects (**Figure 2**).

### 5.1 Development of antibiotics with “special functions.”

Since the systemic use of antibiotics generally fails to completely eradicate bacteria within the OLCN (Theuretzbacher et al., 2020), researchers have evaluated the local bactericidal concentration of “special” antibiotics in the bone infection area. Adjei-Sowah, E. et al. used fluorescent bisphosphonate probes in a murine *S. aureus* (UAMS-1 and USA300LAC)*-infected* tibia model and indicated labeling of the bone surface near the bacteria. They proposed that bisphosphonate-conjugated antibiotics (BCA) application and a “targeting and releasing” can better deliver antibiotics to the site of bone infection. Bisphosphonic acid and hydroxy-bisphosphonate-conjugated antibiotics of sitafloxacin and tedizolid were synthesized using hydroxyphenyl and aminophenyl carbamates, respectively. These conjugates indicated significant serum stability. Furthermore, Sitafloxacin O-phenyl carbamate BCA successfully eradicated static biofilms, whereas the less-stable tedizolid N-phenyl carbamate BCA had limited efficacy against Methicillin-Sensitive *S. aureus* (MSSA) and MRSA. These results prove that BCA can efficiently eradicate *S. aureus* biofilms on the OLCN bone surface and support the *in-vivo* drug development of sitafloxacin BCA (Adjei-Sowah et al., 2021) (**Figure 2A**). Ren Y et al. also employed the “targeting and releasing” kinetic approach to propose the design of two bone-targeting bisphosphonate-conjugated antibiotics, bisphosphonate-conjugated sitafloxacin (BCS) and hydroxy-bisphosphonate-conjugated sitafloxacin (HBCS). They evaluated the *in-vivo* efficacy of BCS and HBCS relative to bisphosphonate, sitafloxacin, and vancomycin in a murine implant-related osteomyelitis model. The vancomycin, sitafloxacin, and placebo groups indicated the presence of autolytic bacteria of colonized *S. aureus* (USA300LAC) in the OLCN of infected tibias of mice, whereas the abundance of most bacteria in the OLCN of infected tibias of BCA-treated mice was relatively low. Compared with the placebo and free-antibiotic controls, BCA also significantly increased the OLCN diameter. These findings support the bone-targeting strategy of BCA to overcome the bi-distribution limitation of standardized antibiotic treatment, which has difficulty accessing the OLCN (Ren et al., 2022).

### 5.2 Development of ideal bone defect filling and repair materials

Chronic bone infections are often accompanied by extensive bone defects. Therefore, it is critical to develop bone defect-filling materials that possess an appropriate elastic modulus to eliminate dead space, exhibit good antibacterial and osteogenic activities, and are degradable. Since the 1960s, antibiotic-loaded bone cement has been the primary choice for the clinical treatment of infected bone defects and was once widely regarded as a standard strategy (Jiranek, 2005; Schwarz et al., 2021) (**Figure 2B**). However, there are several limitations to antibiotic-loaded bone cement, such as incompletely eradicating biofilm-associated bacteria, not repairing bone defects, and being non-biodegradable, necessitating a second-stage surgery. Biomaterials for treating bone infection defects mainly fall into the following categories: bioactive glass (with intrinsic antibacterial, osteoconductive, and angiogenic properties), antibiotic-impregnated calcium-based bone substitutes and calcium phosphates (whose chemical and crystal structures are similar to the inorganic components of bone and have a good infection control rate, yet attention should be paid to the degradation rates of different materials), and polymers (natural polymers such as collagen, chitosan, and gelatin are hydrophobic and highly biocompatible, with the main drawback of being prone to trigger immune responses. Synthetic polymers have a longer shelf life, uniform microstructure, and good mechanical strength, but they can be divided into degradable and non-degradable types) (Jiang et al., 2024). For example, a BSG + Fe^3^O^4^ magnetic scaffold was developed based on the bactericidal properties of magnetic Fe^3^O^4^ nanoparticles in an alternating magnetic field, combined with the excellent osteogenic induction and immunomodulatory properties of borosilicate bioactive glass (BSG), (**Figure 2C**). The antibacterial and osteogenic properties of the BSG + Fe^3^O^4^ magnetic scaffold against *S. aureus* (ATCC29213) bone infections were evaluated *in vitro* and *in vivo*. The results showed that the BSG + 5% Fe^3^O^4^ magnetic scaffold enhanced the osteogenesis of mesenchymal stem cells (MSCs) and promoted the polarization of macrophages to the M2 type *in vitro*. The rabbit implant-related *S. aureus* bone infection model also confirmed that this magnetic scaffold had improved antibacterial effects at the implantation site, effectively controlling the *Staphylococcus* abscess communities and *S. aureus* within the OLCN. Furthermore, it also promotes new bone formation around the primary infected site, thereby effectively addressing the treatment challenges in the infected bone defects. Moreover, the degradation rate of this bioactive scaffold can effectively match the bone formation rate (Jin et al., 2024).

Other than antibiotic-loaded bone cement, autologous or allogeneic bone is also the optimal source for repairing infected bone defects because of its good osteogenic activity. However, the clinical application of autologous or allogeneic bone is often restricted by several factors, such as donor shortage, discomfort at the bone-harvesting site, infection, and bleeding (Mitra et al., 2017; McNeill et al., 2020). Furthermore, it cannot achieve local anti-infection simultaneously. Therefore, the development of novel bone-repair materials with excellent mechanical properties, osteogenic activity, and local anti-infection capabilities has become a research hotspot. With the rapid advancements in cell technology, biomimetic materials, and microsurgical techniques, the development of tissue-engineered bone (TEB) has significantly progressed and can also be employed as a bone-repair material to treat infected bone defects (Qin et al., 2024). The TEB comprises scaffold materials, seed cells, and cytokines (**Figure 2D**). The scaffold material is the core component of TEB because all other components are loaded onto the scaffold to function. Based on the basic mechanical support requirements, an ideal scaffold system for bone infection repair should provide infection treatment with bone defect regeneration. The composite scaffold systems for infected bone defect repair are categorized into hydrogel scaffolds and solid bone tissue substitutes. The hydrogel scaffolds are loaded with stem cells, growth factors, and nanoparticles, whereas the solid bone tissue substitutes mainly include growth factors, nanoparticles, exosomes, and stem cells (Qin et al., 2024). In the treatment of bone infections, hydrogel scaffolds can load bioactive molecules such as antibiotics and growth factors. These molecules can exert their biological effects through diffusion and osmosis, thus effectively inhibiting the growth and reproduction of bacteria. At the same time, they can promote the repair and regeneration of bone tissue and alleviate bone infections. For example, studies have shown that hydrogel scaffolds loaded with rat bone marrow mesenchymal stem cells (rBMSCs) (Bastami et al., 2024) and adipose-derived stem cells (ASCs) (Tang et al., 2020) have excellent adhesion, proliferation, and differentiation abilities, and significant new bone regeneration ability. Hydrogel scaffolds loaded with growth factors such as bone morphogenetic protein (BMP) and stromal cell-derived factor-1 (SDF-1) enhance bone regeneration (Ratanavaraporn et al., 2011). Hydrogel scaffolds loaded with nanoparticles can achieve a gradual and sustained release of antibiotics by incorporating antibiotics into the hydrogel and then embedding the hydrogel in the nanoparticles. They have good injectability and antibacterial activity, thus achieving the purpose of preventing bone infections (Posadowska et al., 2016; Loebel et al., 2017; Wassif et al., 2024). Solid scaffolds, on the other hand, have advantages such as stable shape, complex structure, and diverse functions. Most importantly, they can provide support and elastic modulus that match the mechanical properties of bone tissue through material optimization. Therefore, in the strategy for treating infectious bone defects, it is also a very good choice to use functional-integrated solid scaffolds for filling and treatment. Most solid implants usually require modification of their surface itself to address issues such as bacterial infections in bone infections (avoiding bacterial adhesion, killing bacteria, and reducing biofilm formation), inflammatory responses, and bone regeneration (Epstein et al., 2011; Vlamakis et al., 2013; Ghosh et al., 2019). However, currently, there are no studies on specifically eradicating *S. aureus* in bone microstructures using TEB methods, and such future research on bone infection treatment *via* TEB technology could focus on loading targeted antibiotics onto matrix materials with different properties and simultaneously choosing appropriate scaffold preparation methods. The treatment goal is to clear bacteria in biofilms and OLCN to promote infection eradication and bone regeneration. This approach can overcome the bottleneck of traditional treatment methods for bone infection and defects, which often require multiple surgeries.

### 5.3 Development of internal fixation materials with anti-infection functions

In the surgical treatment of infected bone defects, after thorough debridement, internal fixators are usually required to firmly fix the bone stumps (or the bone defect area) for good mechanical stability, which is conducive to bone repair. However, the internal fixator’s surface is a prone site for bacterial biofilm formation (Zimmerli et al., 2004; Yousif et al., 2015), which acts as a barrier, creating a stable environment for bacterial growth. The biofilm protects bacterial cells from extreme conditions such as high temperatures, nutrient deficiency, pH changes, and antibiotics. Furthermore, systemic antibiotics often fail to completely eradicate bacteria within the biofilms and OLCN (Theuretzbacher et al., 2020). Therefore, it is essential to elucidate methods that allow internal fixators to locally release sufficient concentrations of antibiotics in the infected area to effectively combat bacteria while minimizing systemic toxicity (Birk et al., 2021; Sayed et al., 2022; Wang et al., 2022). Therefore, various local antibacterial release systems have been developed to treat bone infections, specifically in titanium alloy implants widely used in orthopedics (Wu et al., 2021; Zhang et al., 2023; Zhou et al., 2023). Previous literature suggests that the acidic environment in a bacterial infection can be employed to develop a pH-responsive antibacterial surface on implants (Chen et al., 2020; Zhang et al., 2023; Zhou M. et al., 2024). Some scholars have successfully synthesized pH-responsive polymethacrylic acid (PMAA)-gated TiO^2^ nanotubes on titanium plates. The PMAA molecules provide an on-demand release of antibacterial peptides through the “swelling-collapse” transition (Chen et al., 2020). Moreover, to improve the nanotube’s stability, a new type of screw was developed by preparing enhanced TiO^2^ nanotubes on titanium screws; it immobilizes PMAA and loads the antibacterial peptide HHC36 (**Figure 2E**). In an acidic infected environment, this novel screw indicated significant pH-responsiveness and enhanced antibacterial effect by the on-demand HHC36 release. The simulated clinical implantation process has indicated that this novel screw can maintain excellent pH-responsive antibacterial performance under mechanical stress (Zhou H. et al., 2024). Further, the research also revealed that the antibacterial peptide HHC36 completely eradicates the residual bacteria in SACs and OLCN (Zhou H. et al., 2024). Altogether, the development of internal fixators with controlled and on-demand sustained release of antibiotics can effectively eradicate bacteria that have invaded the OLCN, providing a new direction for the prevention and treatment of recurrent chronic bone infections.

## 6 Discussion and foresight

The invasion of bacteria into the OLCN of bone tissue is the basis of COM pathology and is crucial for clinical treatment, especially for recurrent osteomyelitis. The three-dimensional structure of OLCN indicates that it is an ideal “refuge” for *S. aureus*. Firstly, osteocytes, the main OLCN components, are enclosed in the bone canaliculus network and send out cellular processes that extend into the canaliculi to connect adjacent osteocytes. *In vitro* studies have indicated that *S. aureus* can invade and survive within osteocytes without causing cell death (Yang et al., 2018). Furthermore, during osteocyte infection, *S. aureus* adapts to the environment by adopting a survival mode similar to that of small-colony variants (SCVs), thereby supporting persistent or occult infections. Clinical case studies have also confirmed significant intracellular colonization of *S. aureus* in osteoblasts and osteocytes in chronically infected bone tissues (Bosse et al., 2005; Sendi et al., 2006) than other cell types, such as macrophages (Garzoni and Kelley, 2011), epithelial cells (Dziewanowska et al., 1999), keratinocytes (Kintarak et al., 2004), and endothelial cells (Edwards et al., 2010). *S. aureus* infection of osteocytes is particularly pathogenic. *S. aureus* intracellularly infects osteoblasts, which, upon differentiation or maturation into osteocytes, serve as a reservoir for long-term bacterial colonization in bone tissue and easily evade the immune system (Alder et al., 2020). Furthermore, *S. aureus* can induce the secretion of osteoclast-related cytokines, which promote pathological bone loss. Moreover, large-sized bacteria like *S. aureus* (1 μm diameter) can change their shape and reduce their volume by nearly half to penetrate the sub-micron-level (0.5 μm diameter) interstitial channel network in the dense bone structure (You et al., 2004; Cai et al., 2022). This allows them to survive for an extended period and evade the surveillance of immune cells. Although bromodeoxyuridine labeling studies on mice have revealed that orally administered small molecules can reach *S. aureus* colonized within the OLCN (Sultan et al., 2019), other studies suggest that the combined use of high-dose local and systemic antibiotics fails to eradicate OLCN invasion by MSSA (Veis and Cassat, 2021) and MRSA (Masters et al., 2020). This might be because their adaptive responses are associated with persistent intracellular survival and SCV formation (Schwarz et al., 2021). Lastly, the small-sized *S. aureus* SCVs that invade and survive within osteocytes may also be secreted into the osteocyte lacunae and canaliculi. The survival of *S. aureus* in such bone microstructures makes them a continuous source of occult infection. Therefore, new treatment methods targeting the morphological changes of *S. aureus* (such as transformation into SCVs) during its invasion of the OLCN should be developed for effective COM treatment.

### 6.1 OLCN is a potential “special” niche for the persistent *S. aureus* SCV survival

*S. aureus* SCVs are a special bacterial phenotype that grows slowly and forms tiny colonies on agar plates. Compared to the normal *S. aureus* colonies, their diameter is significantly smaller (the colony size is only approximately one-tenth of wild-type bacterial colonies) (Proctor et al., 2006; Nguyen et al., 2009; Garcia et al., 2013). They were first reported approximately 100 years ago and were described as naturally occurring populations, typically the “G” type or “dwarf” colonies of many bacterial species, including *S. aureus* (Eiff et al., 2006; Proctor et al., 2006). Due to the special *S. aureus* SCVs morphology and significantly reduced metabolic rate, they have markedly reduced sensitivity to antibiotics, making their detection and identification difficult in routine testing and culturing conditions. As a result, they are associated with persistent and recurrent infections, which have been extensively studied (Proctor et al., 2006).

The identification of factors that induce the formation of *S. aureus* SCVs is significant for preventing chronic *S. aureus* bone infections, specifically for reducing the entry of *S. aureus* into the OLCN and its survival as SCVs. Some early studies considered SCVs as gonidial mutants or “G” forms that develop within specific mother cells under adverse conditions. They are extremely small and may represent the primitive stage of the bacterial life cycle (Swingle, 1935; Wise and Spink, 1954). Recent reports isolated “dwarf” colonies from animals and humans after antibiotic treatment (Goudie and Goudie, 1955; Sompolinsky et al., 1974). Previous literature on SCVs of different bacterial species has indicated that SCV formation is a natural survival mechanism for many bacteria and is mostly generated under selective conditions such as antibiotic treatment, cold stress, disinfectant exposure, or within eukaryotic cells (Atalla et al., 2011). Therefore, it is essential to further explore the inducing factors and underlying mechanisms of this bacterial phenotype. The small volume of SCVs may help *S. aureus* enter bone lacunae and canaliculi from osteocytes. The identification of the relationship between the formation of *S. aureus* SCVs and OLCN invasion may help in the development of targeted treatment strategies for the SCV phenotype. This mechanism can reduce the bacterial load in the OLCN, thereby decreasing the incidence of persistent infections.

### 6.2 Sub-minimum inhibitory concentration antibiotics may play a crucial role in SCV formation and OLCN invasion

#### 6.2.1 Clinically, the antibiotics for treating COM caused by *S. aureus* are primarily administered locally or systemically

The commonly used systemic antibiotics include β-lactams, clindamycin, and fluoroquinolones (Tuchscherr et al., 2016). The minimum inhibitory concentration (MIC) is defined as the lowest concentration of an antibiotic that can inhibit the pathogenic bacteria growth in the culture medium after 18 to 24 hours of *in vitro* bacterial culture. MIC measures the antibacterial activity of antimicrobial agents. Theoretically, when the drug concentration reaches the MIC, bacterial growth and reproduction will be inhibited. *In vivo*, if the drug concentration at the infection site is continuously maintained at or above the MIC, and the bacteria becomes sensitive to the drug, thereby gradually relieving the patient’s symptoms, such as fever, redness, swelling, and pain. Furthermore, inflammatory indicators, including white blood cell (WBC) count, C-reactive protein (CRP), erythrocyte sedimentation rate (ESR), and procalcitonin (PCT), also gradually decrease to the normal range. Moreover, the body’s immune system slowly eliminates the inhibited bacteria, thus achieving the goal of the treatment. However, clinically, the blood and local drug concentrations at the infection site cannot reach the MIC due to various reasons, thus forming sub-minimum inhibitory concentration (sub-MIC). Antibiotics at sub-MIC have no inhibitory effect on bacterial growth and induce metabolic and morphological changes in the bacteria. To cope with the drug pressure, bacteria produce some proteins or enzymes to resist the drug’s action and maintain their survival, which may lead to drug resistance. The literature points out that the action of antibiotics at sub-MIC can promote three main types of morphological changes in *S. aureus*, including cell morphological deformation, cell wall component alteration, and cell wall rupture (Braga et al., 1997; Mette et al., 2013; Guanghui et al., 2014). In 2021, Juan Chen et al. reviewed the impact of sub-MIC antibiotics on *S. aureus* morphology (Juan et al., 2021). They evaluated the impact of antibiotics, including dicloxacillin, cefodizime, cefotaxime, ceftriaxone, cefodizime, ciprofloxacin (CFX), berberine, tetracycline, thioridazine, and ceftobiprole at sub-MIC on the morphological variations of *S. aureus.* They revealed varying degrees of morphological alteration, such as increased volume, irregular deformation, damaged and ruptured cell walls, increased cell membrane permeability, and decreased adhesiveness. Moreover, they showed that antibiotics at different sub-MIC may have different effects on *S. aureus’s* cell morphology (Juan et al., 2021). Furthermore, certain sub-MIC antibiotics (such as methicillin and cefoxitin) can weaken the cell wall of *S. aureus* by binding to PBPs (Shang et al., 2019). Therefore, it was speculated that *S. aureus* morphological variations are caused by sub-MIC antibiotics, and their binding with PBPs may be associated with the invasion of *S. aureus* into the OLCN. However, further exploration and verification are warranted.

#### 6.2.2 The sub-MIC antibiotics also affect *S. aureus* adhesion and invasion

Research has shown that the pathogenicity of *S. aureus* is markedly linked with its ability to adhere to host cells or the ECM (Josse et al., 2017). Similarly, *S. aureus’s* entrance into osteocytes and canaliculi is important for its adhesion and colonization in the OLCN. The adhesion molecules responsible for *S. aureus* adhesion mainly include clumping factors A and B, staphylococcal fibrinogen-binding proteins A and B, serine-aspartate repeat-containing protein D, *etc.* (Zheng et al., 2020). Juan Chen et al. revealed that the effects of sub-MIC antibiotics on the adhesion and invasion of *S. aureus* may vary depending on the bacterial strains and host cell models used (Juan et al., 2021). Furthermore, several studies have indicated that the effects of sub-MIC antibiotics on the adhesion of different *S. aureus* strains also vary. Some strains indicate an enhanced adhesion effect, while others may show decreased adhesion ability. Further in-depth investigation of the effects of sub-MIC antibiotics on the adhesion and invasion of *S. aureus* is crucial for the proper application of antibiotics and the reduction of the bacterial load in the OLCN.

#### 6.2.3 Local application of antibiotics in the bone infection site is also a critical means and classic strategy for treating COM caused by *S. aureus*

The advent of antibiotic-loaded bone cement is advantageous for repairing infected bone defects by eliminating dead spaces and promoting antibacterial effects locally. Therefore, it is widely used for the clinical treatment of infected bone defects. Antibacterial drugs in bone cement should have thermal stability, water-solubility, a broad antibacterial spectrum, high antibacterial efficacy, few naturally resistant bacteria, less binding to proteins, low allergenicity, no systemic toxic reactions, and minimal impact on the mechanical strength of the bone cement. Currently, the antibiotics employed in antibiotic-loaded bone cement depend on the types of infected microorganisms and primarily include gentamicin, tobramycin, and vancomycin powder (Saeed et al., 2019). However, there are certain disadvantages of using antibiotic-loaded bone cement such as when the local antibiotic concentration fails to reach the MICs, it promotes the formation of SCVs. L. Tuchscherr et al. showed that *in vitro*, rifampicin can almost clear infected osteoblasts in acute and chronic phases. Whereas low-concentration of gentamicin, moxifloxacin, and clindamycin can induce SCV formation. Moreover, gentamicin, fosfomycin, and clindamycin promote the rapid formation of SCVs within osteoblasts, which may result in chronic infections. The acute and chronic mouse osteomyelitis model revealed that during the acute phase, only rifampicin significantly reduced the bacterial load in bone tissue, while cefuroxime and gentamicin showed poor effects. Moreover, gentamicin induces the formation of SCVs (Tuchscherr et al., 2016), thus confirming that gentamicin is a potent inducer of SCVs.

Based on the above three aspects, about 70% of patients receiving long-term antibiotic treatment may develop *S. aureus* SCVs infections (Melter and Radojevič, 2010). Therefore, the role of sub-MIC antibiotics (especially those like gentamicin) in promoting the formation of *S. aureus* SCVs should not be ignored, as it significantly impacts the promotion of persistent and recurrent infections in clinical practice. Furthermore, the SCVs-inducing activity of antibiotics such as gentamicin should be considered specifically during the treatment of infected bone defects. Since antibiotic-loaded bone cement is often applied locally as a surface coating for internal fixators or a filler for defect areas, antibiotics like gentamicin should be used in higher concentrations to achieve a bactericidal effect. Therefore, since sub-MIC gentamicin can induce SCV formation, its doses in host cells, bone defect areas, or bone tissues should be monitored. The rapidly formed SCVs promote prolonged bacterial survival in the host. The persistent survival, adhesion, and colonization of *S. aureus* in osteocytes and canaliculi are the main causes of COM. In the future, the relationship and mechanism of action between *S. aureus* SCVs induced by sub-MIC antibiotics and OLCN invasion should be comprehensively investigated. Developing a bone-targeted antibiotic with a local concentration that can exceed the MIC is significant for the treatment of deep bone infections.

### 6.3 OLCN may provide a suitable environment for the survival of *S. aureus*

The relatively closed OLCN microenvironment provides stable metabolic support and suitable survival conditions for pathogenic bacteria (Moriishi and Komori, 2022; Evans et al., 2024). The pH of OLCN plays a crucial role in the osteocyte’s function and also provides favorable conditions for bacteria invasion. Recently, several researchers from the bone biology field have investigated the pH of the OLCN microenvironment. The literature suggests that under normal physiological conditions, the OLCN’s pH remains relatively stable and slightly alkaline, around 7.4 - 7.6. The metabolic activities of osteocytes and the composition of the surrounding tissue fluid jointly maintain the pH in the OLCN. During metabolic processes such as aerobic respiration, osteocytes produce carbon dioxide, which combines with water to form carbonic acid, which in turn dissociates to produce hydrogen and bicarbonate ions. The hydrogen ion concentration is finely regulated by the intracellular buffer system and ion transport mechanisms, keeping the OLCN’s pH within an appropriate range. For example, carbonic anhydrase in osteocytes can catalyze the reaction between carbon dioxide and water, whereas the cell membrane transport proteins and ion channels transport bicarbonate and hydrogen ions across the transmembrane, thereby maintaining the intra- and inter-cellular acid-base balance. In infectious diseases such as implant-related *S. aureus* osteomyelitis, the metabolic products of bacteria and the inflammatory response may decrease OLCN’s pH. *S. aureus* produces acidic substances, such as lactic acid and acetic acid, during metabolism. The local accumulation of these acidic metabolic products can reduce OLCN’s pH to 6.5 or even lower. Furthermore, inflammatory mediators such as TNF-α and IL-1 released by inflammatory cells can stimulate osteoclasts and promote bone resorption. Osteoclasts secrete acidic substances, including hydrogen ions, during bone resorption, which further exacerbates the acidic environment in the OLCN (Zhou H. et al., 2024). The pH changes in OLCN’s environment affect the normal functions of osteocytes. It may promote bacterial survival and reproduction, forming a vicious cycle and exacerbating the infection. In an acidic environment, osteocyte’s metabolic activities are inhibited, their ability to synthesize and secrete bone matrix reduces, and their survival is threatened. For bacteria, although a lower pH environment can inhibit their growth to a certain extent, some bacteria, such as *S. aureus*, have acid tolerance and can not only survive but also cause persistent infections in the acidic OLCN. For instance, the acidic environment (pH 3-5) of bone resorption lacunae may inhibit the functions of complement and antimicrobial peptides. It has been observed that in pH < 6, the synthesis of complement C3 and C5 convertases is inhibited, which weakens the complement cascade reaction, including C3b deposition and C5a generation (Hany Ibrahim et al., 2015). Antimicrobial peptides, defensins, rely on their cationic properties to bind to the bacterial membrane and form pores. An acidic environment alters the cationic characteristics of these peptides and weakens their bacterial cell membrane binding ability, thus reducing their bactericidal effect (Mahmoud H et al., 2014). Similarly, the acidic environment may enhance pathogenic bacteria’s tolerance to acid stress by inducing the outer-membrane proteins of pathogenic bacteria (such as ClfA in *S. aureus*) or lipid-metabolic pathways. The hypoxic environment in the bone microstructure may induce a dormant state in pathogenic bacteria, which reduces energy consumption and enhances their tolerance to the immune system to adapt to the hypoxic environment. The literature also revealed that the acidic OLCN environment reduces the body’s immune function, decreases the bactericidal effect of antimicrobial peptides, and provides a favorable survival environment for pathogenic bacteria such as *S. aureus*. There is research on the association between the pH environment of the OLCN and *S. aureus;* however, several factors require further investigation. In future research, the whole-genome expression profiles and proteomic changes of *S. aureus* under different pH OLCN environments should be analyzed to comprehensively understand its survival-adaptation and pathogenic mechanisms. Further, more precise and efficient pH-regulation technologies and combination therapies, as well as pre-clinical and clinical trials, are required to translate the research results into clinical applications and provide better treatment options for bone infection patients.

## 7 Conclusion

In conclusion, both clinical case reports and some animal model studies have indicated that the invasion of *S. aureus* into OLCN is one of the important mechanisms that cannot be ignored for its immune escape and the persistence of bone infections, which are difficult to heal. Existing studies have shown that various mechanisms mainly involving the components of the cell wall of *S. aureus* are involved in its morphological variation and promote its invasion into OLCN. In view of this special mechanism of *S. aureus* entering OLCN, innovative strategies and techniques for treating bone infections have been proposed from three aspects, namely, improving antibiotics, developing new bone defect repair materials, and implant materials. Meanwhile, the interaction among osteocytes, antibiotics at sub-MIC, and the formation of SCVs may play an important role in the invasion of the bone microstructure by *S. aureus*. This not only provides a new perspective for understanding chronic bone infections but also opens up new avenues for their treatment, and promotes the research in this field to develop in a more precise direction. Through this comprehensive perspective, we earnestly expect to achieve significant breakthroughs in the treatment of bone infections in the future, thereby effectively improving the treatment outcomes and quality of life of patients.
